# Supplementary figures and images for: Colour and motion affect a dune wasp’s ability to detect its cryptic spider predators
Source: Sci Rep. 2021 Jul 29;11:15442. doi: 10.1038/s41598-021-94926-7 (PMC8322161; doi:10.1038/s41598-021-94926-7)

## Spider on the side

2 cm

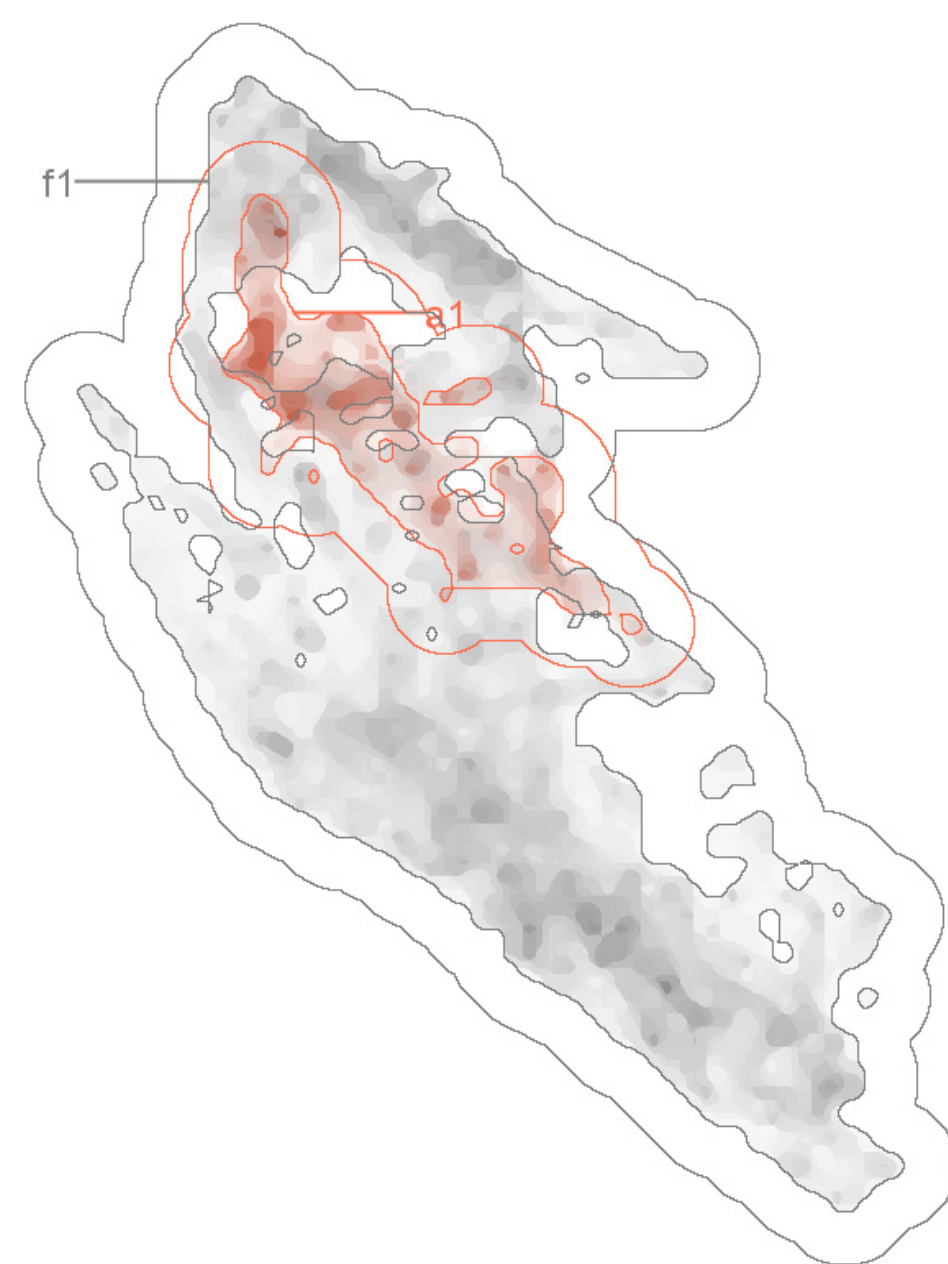

5 cm

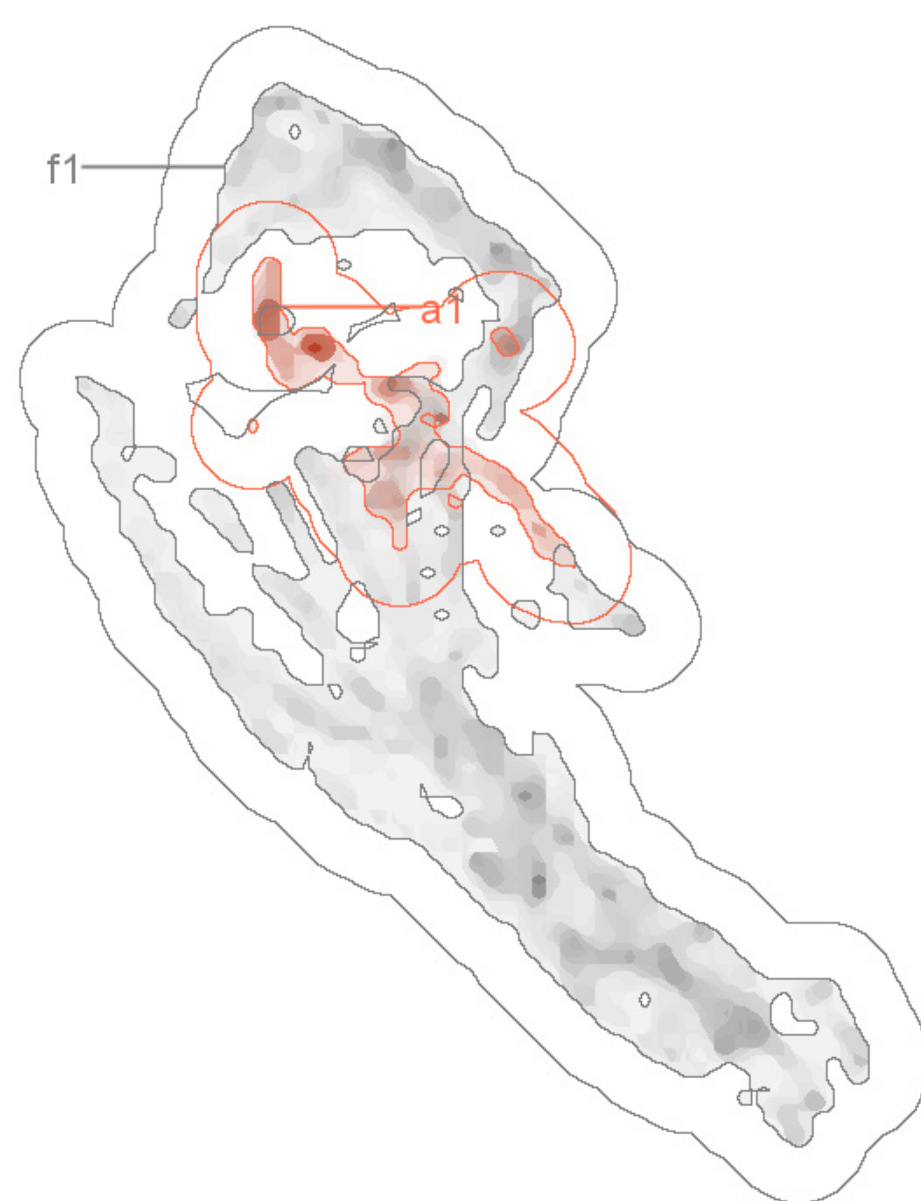

10 cm

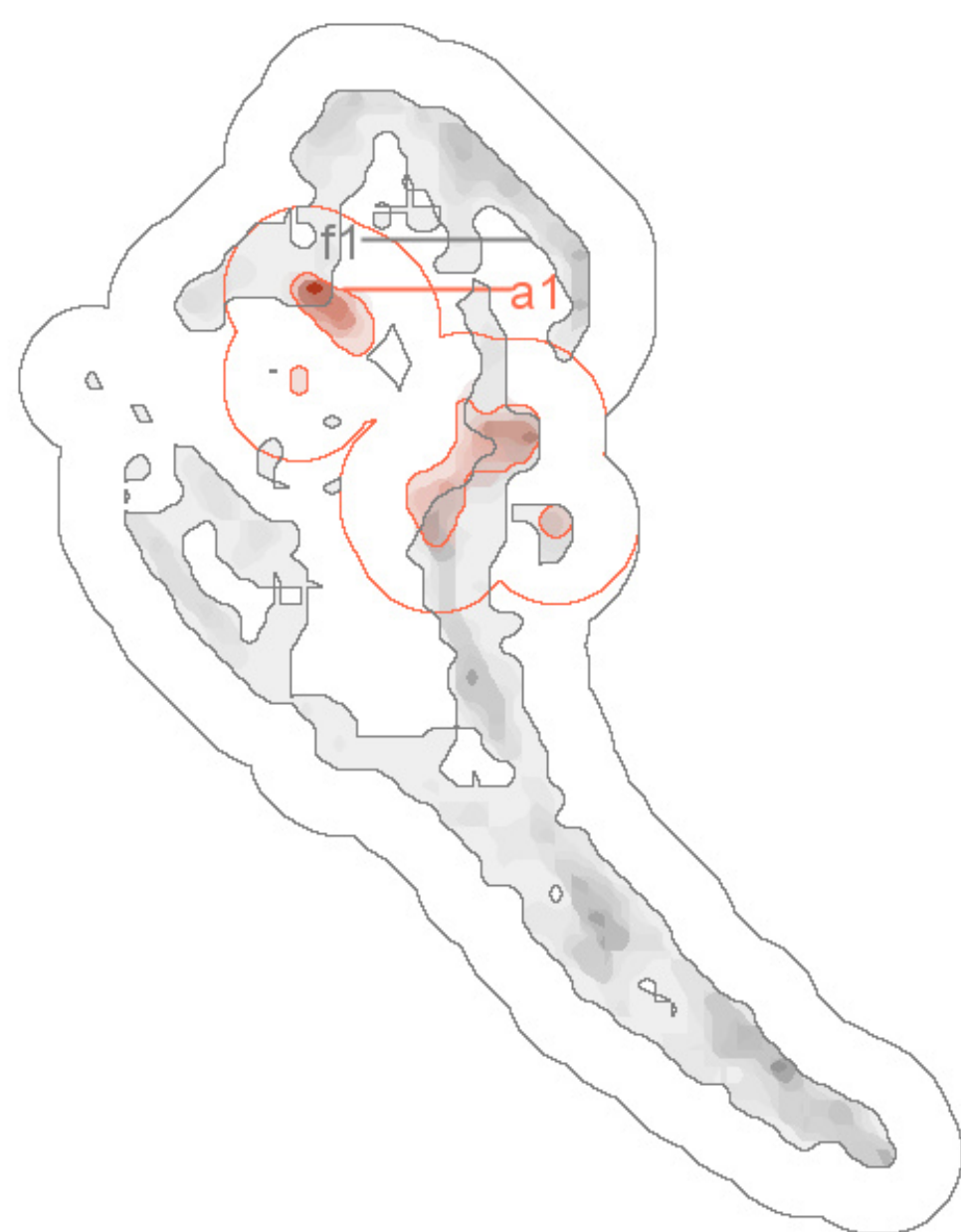

15 cm

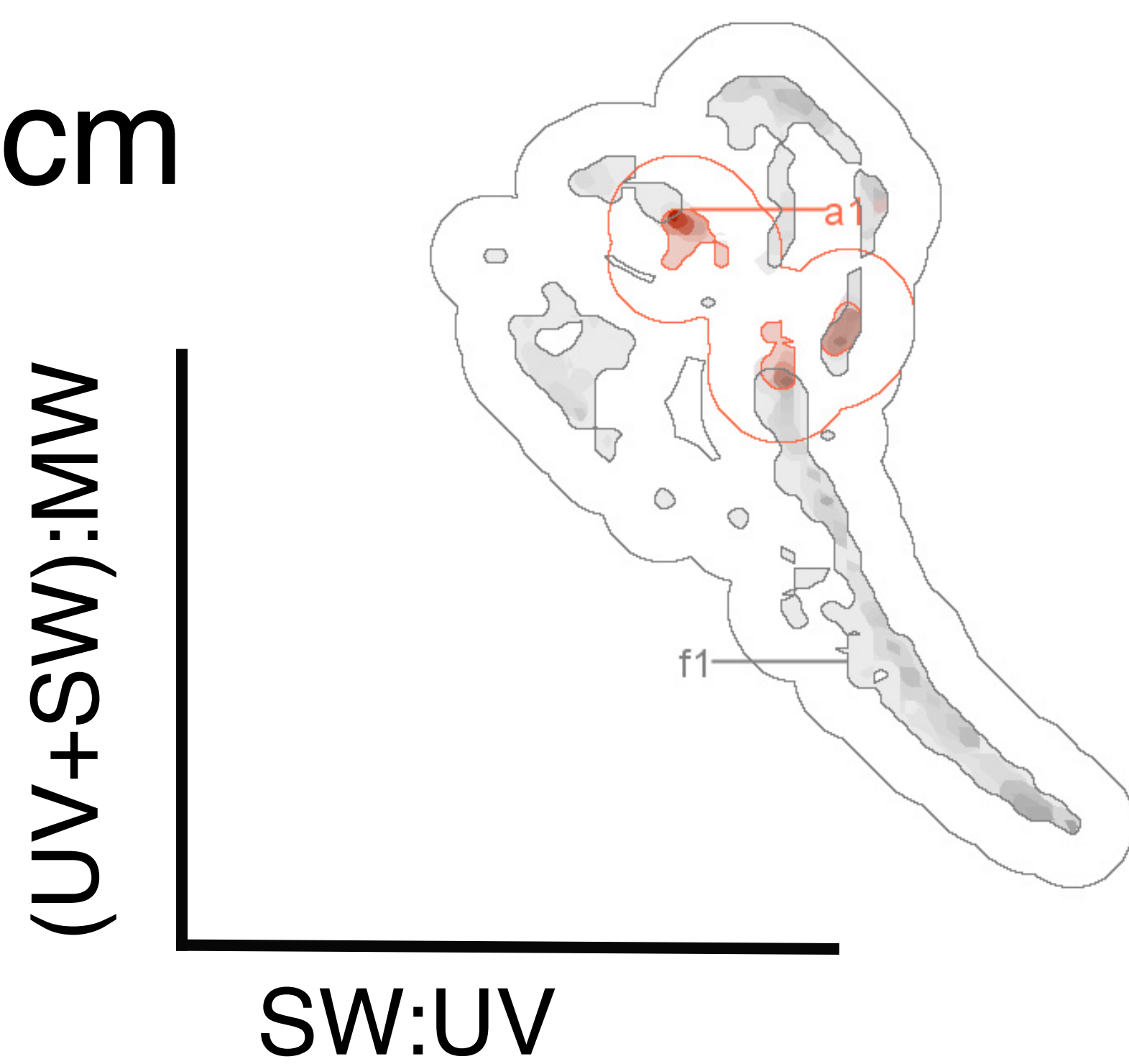

## Spider on the top

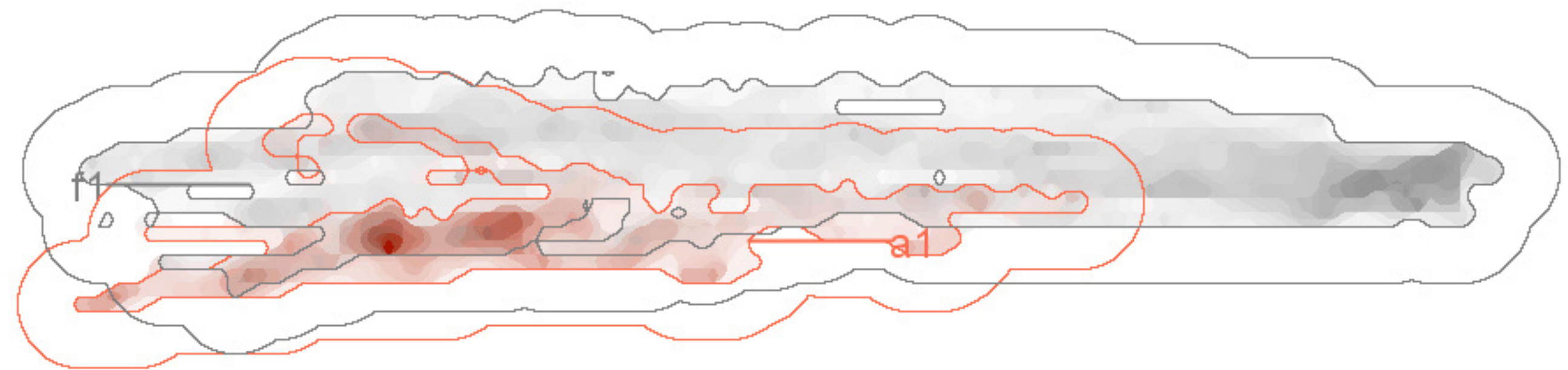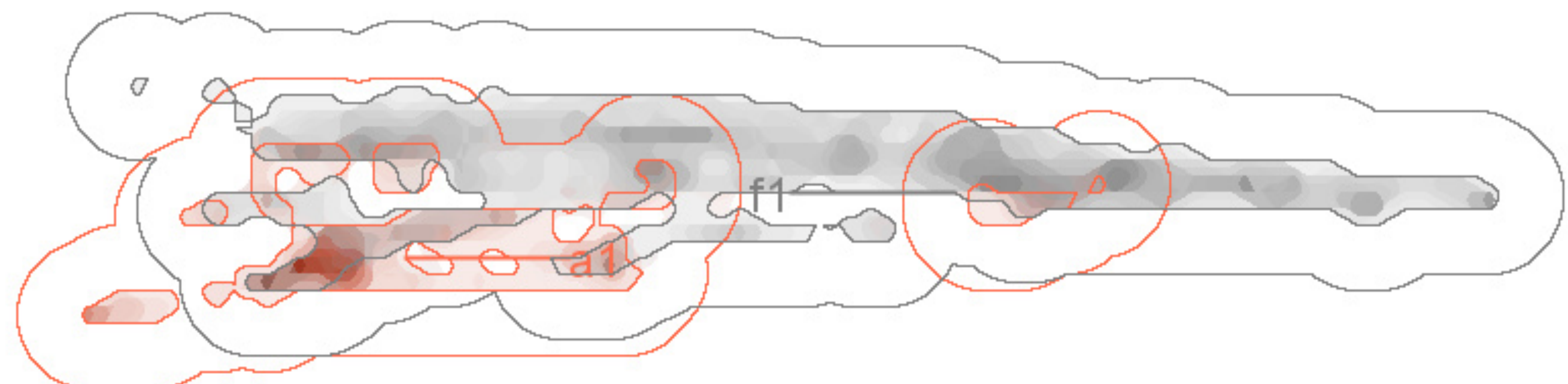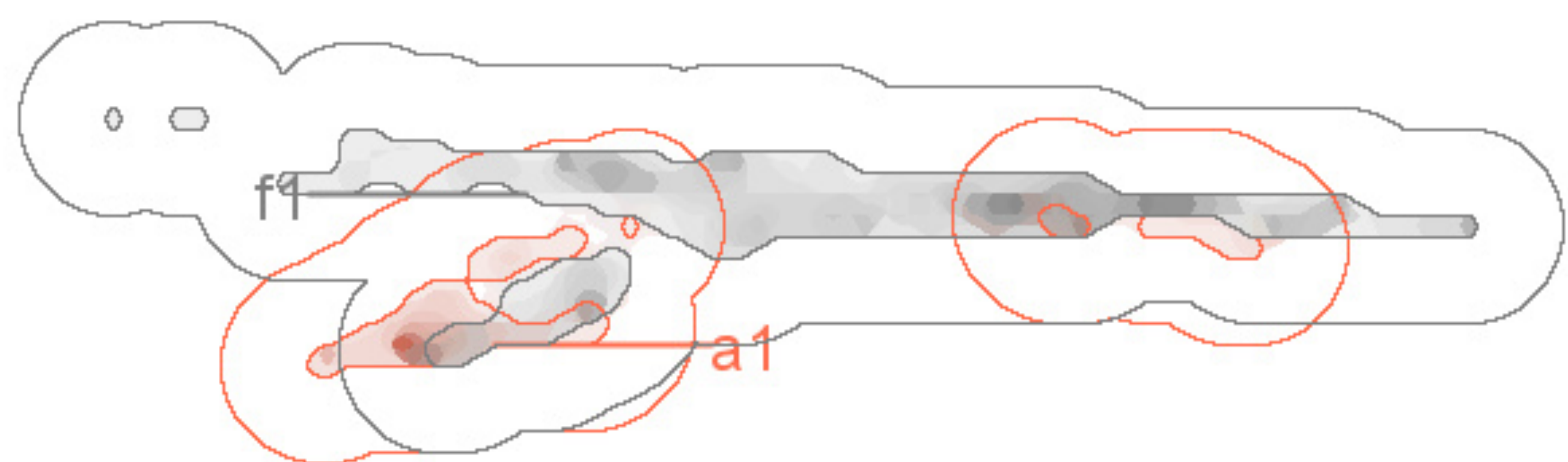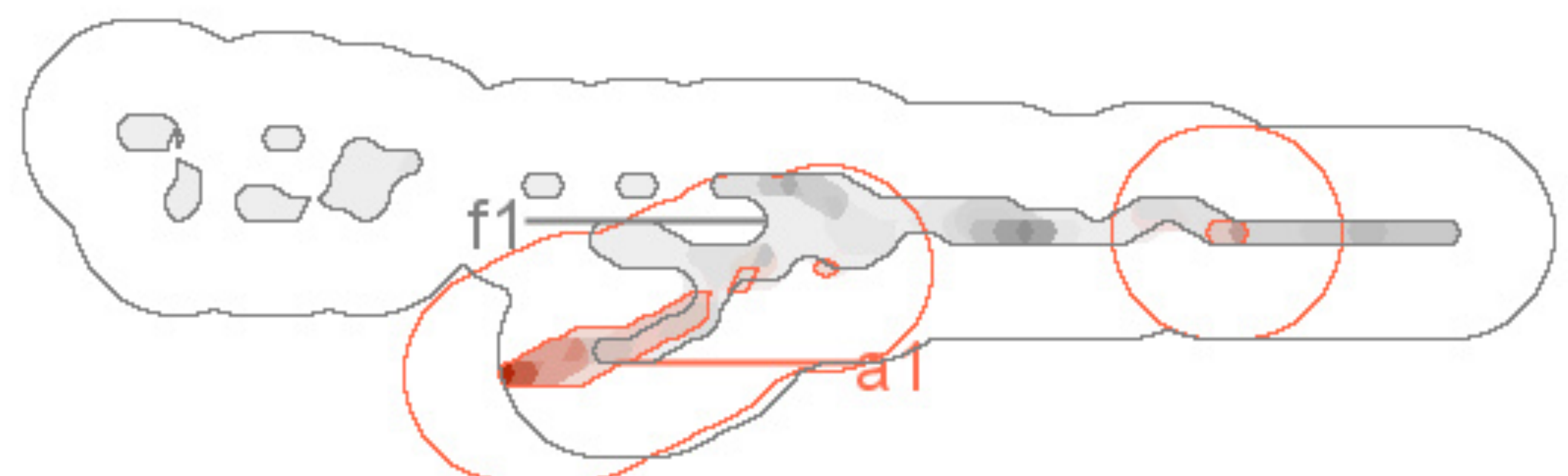

Supplement: Supplementary file 1 — Supplementary Information 1. [file 41598_2021_94926_MOESM1_ESM.pdf]

— Moving  
— Still

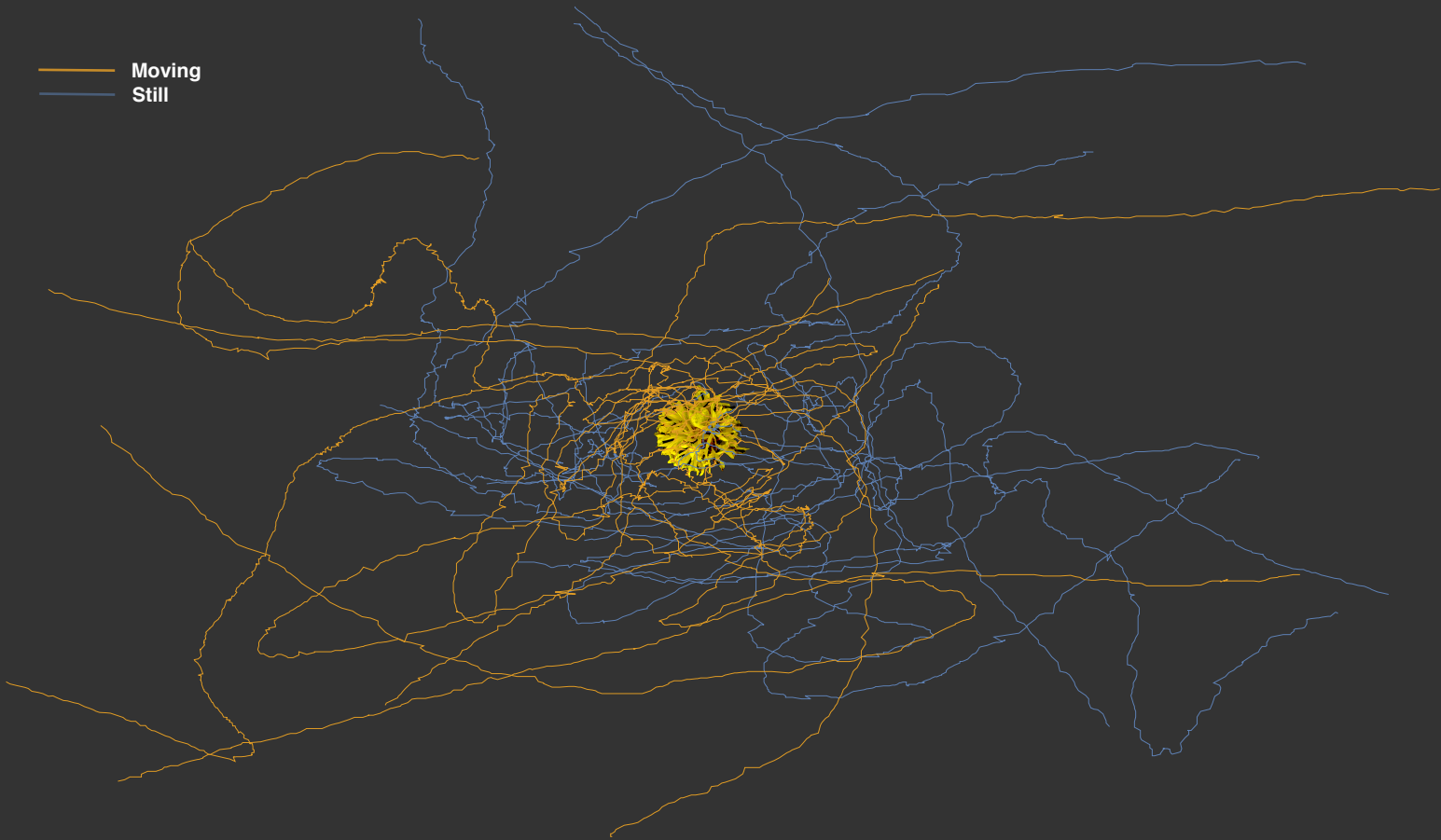

Supplement: Supplementary file 3 — Supplementary Information 2. [file 41598_2021_94926_MOESM3_ESM.pdf]

Figure S4

Dendrogram of DTW unsupervised classification of distance profiles

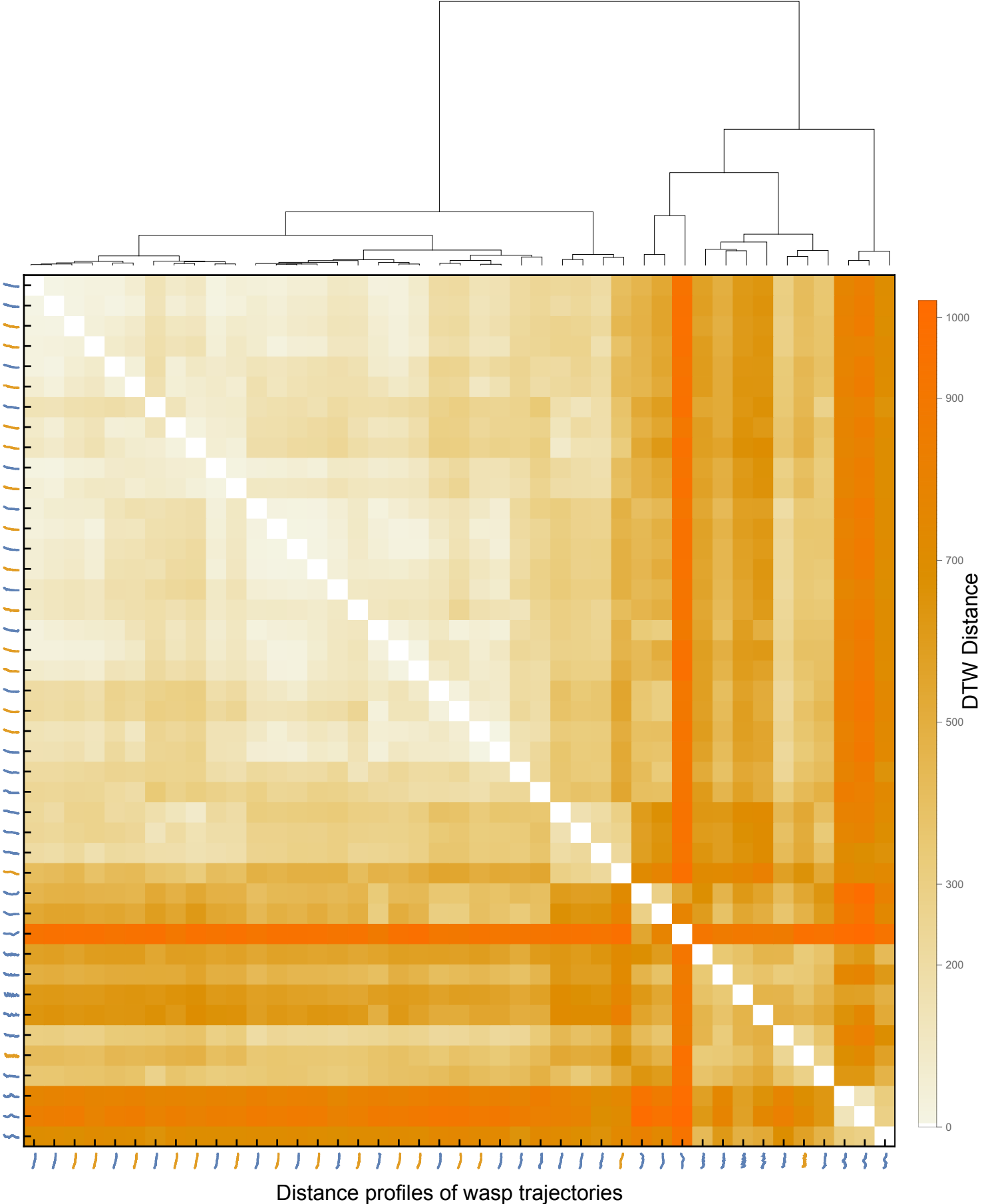

Supplement: Supplementary file 4 — Supplementary Information 3. [file 41598_2021_94926_MOESM4_ESM.pdf]
