# Supplementary material for: Colour and motion affect a dune wasp’s ability to detect its cryptic spider predators
Source: Sci Rep. 2021 Jul 29;11:15442. doi: 10.1038/s41598-021-94926-7 (PMC8322161; doi:10.1038/s41598-021-94926-7)
Supplement: Supplementary file 7 — Supplementary Legends 4. [file 41598_2021_94926_MOESM7_ESM.pdf]

Supplementary files
S1: Colour maps that show the degree of overlap between the spider (a1), the flowerhead (f1) using the Hymenopteran colour space. See text for details.
S2: A sample video showing a wasp approaching an unoccupied still flowerhead.
S3: All trajectories of wasp approaches to Moving (orange lines) and Still (blue lines) flowers. Note that the flowerhead is for illustrative purposes and not to scale.
S4: Distance matrix of the Dynamic Time Warp based unsupervised classification of distance profile of wasp trajectories.
S5: Sample movie showing point when wasp made a decision to avoid the flower.
S6: Sample movie showing point when wasp made a decision to land on the flower.
